# Light‐ and Field‐Controlled Diffusion, Ejection, Flow and Collection of Liquid at a Nanoporous Liquid Crystal Membrane

**DOI:** 10.1002/anie.202207468

**Published:** 2022-07-19

**Authors:** Yuanyuan Zhan, Serena Calierno, Jacques Peixoto, Lars Mitzer, Dirk J. Broer, Danqing Liu

**Affiliations:** ^1^ Department of Chemical Engineering and Chemistry Eindhoven University of Technology Groene Loper 3 5612 AE Eindhoven The Netherlands; ^2^ Institute for Complex Molecular Systems (ICMS) Eindhoven University of Technology Groene Loper 3 5612 AE Eindhoven The Netherlands; ^3^ Department of Chemical Engineering University of Naples Federico II Corso Umberto I, 40 80138, NA Napoli Italy; ^4^ Joint Research Lab of Devices Integrated Responsive Materials South China Normal University Guangzhou 510006 China

**Keywords:** Light and Electrical Response, Liquid Crystal Polymer Network, Liquid Reallocation, Liquid Secretion, Porous Coating

## Abstract

Liquid manipulation at solid surfaces has attracted plenty of interest yet most of them are limited to one or two direction(s), while transport in three dimensions is largely unexplored. Here, we demonstrate three‐dimensionally steered dynamic liquid mobility at nanoporous liquid crystal polymer coatings. To this end, we orchestrate liquid motion via sequential triggers of light and/or electric field. Upon a primary flood exposure to UV light, liquid is ejected globally over the entire coating surfaces. We further reallocate the secreted liquid by applying a secondary electric field stimulus. By doing so, the liquid is transported and collected at pre‐set positions as determined by the electrode positions. We further monitor this process in real‐time and perform precise analysis. Interestingly, when applying those two triggers simultaneously, we discover a UV‐gated liquid‐release effect, which decreases threshold voltage as well as threshold frequency.

## Graphical abstract

Three‐dimensionally steered liquid transport at nanoporous liquid crystal polymer coatings is realized by sequential UV exposure and electric field application. UV induces through‐thickness unidirectional liquid secretion. The subsequently applied electric field reallocates the ejected liquid to predesigned locations. Interestingly, UV‐gated liquid transport is revealed, which dramatically decreases both threshold voltage and threshold frequency.

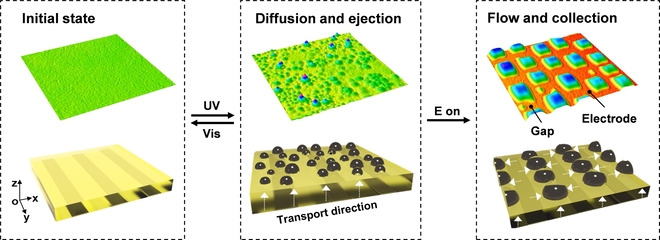

Benefited from various creatures in nature, where numerous examples showcase liquid manipulation behaviours at solid surfaces, material scientists are widely exploring biomimetic surfaces to control liquid motion. In particular, liquid mobility has been extensively investigated at polymer interfaces.^[1]^ Those synthetic coatings tailored with unique surface chemistry and delicate surface structures are endowed with a considerable capability of manipulating liquid.^[2]^ Principles, materials, and fabrication techniques have been developed.^[3]^ Various applications, including self‐cleaning, water collection, no‐loss transport of microdroplets, cargo release, and fouling resistance, among others, are substantially exploited.^[4]^

Recently, we proposed a novel approach to manipulating liquid at self‐contained porous coating surfaces.^[5]^ These dynamic surfaces are composed of liquid crystal polymer networks (LCNs). LCNs possess unusual anisotropic optical and dielectric properties and have been extensively investigated and utilized in display industries.^[6]^ In the past decades, they have been greatly promoted in the field of soft robotics and actuators.^[7]^ Lately, we have used these LCNs for on‐demand through‐thickness liquid secretion and up‐taking at coatings. The liquid can be released either globally or locally by carefully designing molecular architecture. An alternative approach is via patterned interdigitated electrodes (IDEs) when an electric field is employed. So far, liquid transport is restricted to the single through‐thickness direction orthogonal to the surface plane. A facile approach to transporting liquids in three dimensions remains challenging.

In this paper, we present a newly developed strategy to manipulate liquid in/at a polymer coating into multiple dimensions, including through‐thickness directional transport and lateral liquid migration. Our coatings are nanoporous liquid crystal networks (LCNs) filled with a liquid crystal fluid that during it formation acts as the porogen forming the pores. The LCN is copolymerized with a light‐responsive azobenzene derivative. The coating is applied on a glass substrate with interdigitated electrodes structure (Figure 1A). Secretion and the subsequent reallocation of the liquid are realized via an application of sequential light and alternating current (AC) field. UV light secretes liquid uniformly at the coating surfaces, which is further laterally transported and collected at predesigned locations by the AC field. These dual triggers can be used sequentially but when used simultaneously they provide a gating effect steering the secretion in time, place and remotely controlled.

**Figure 1:**
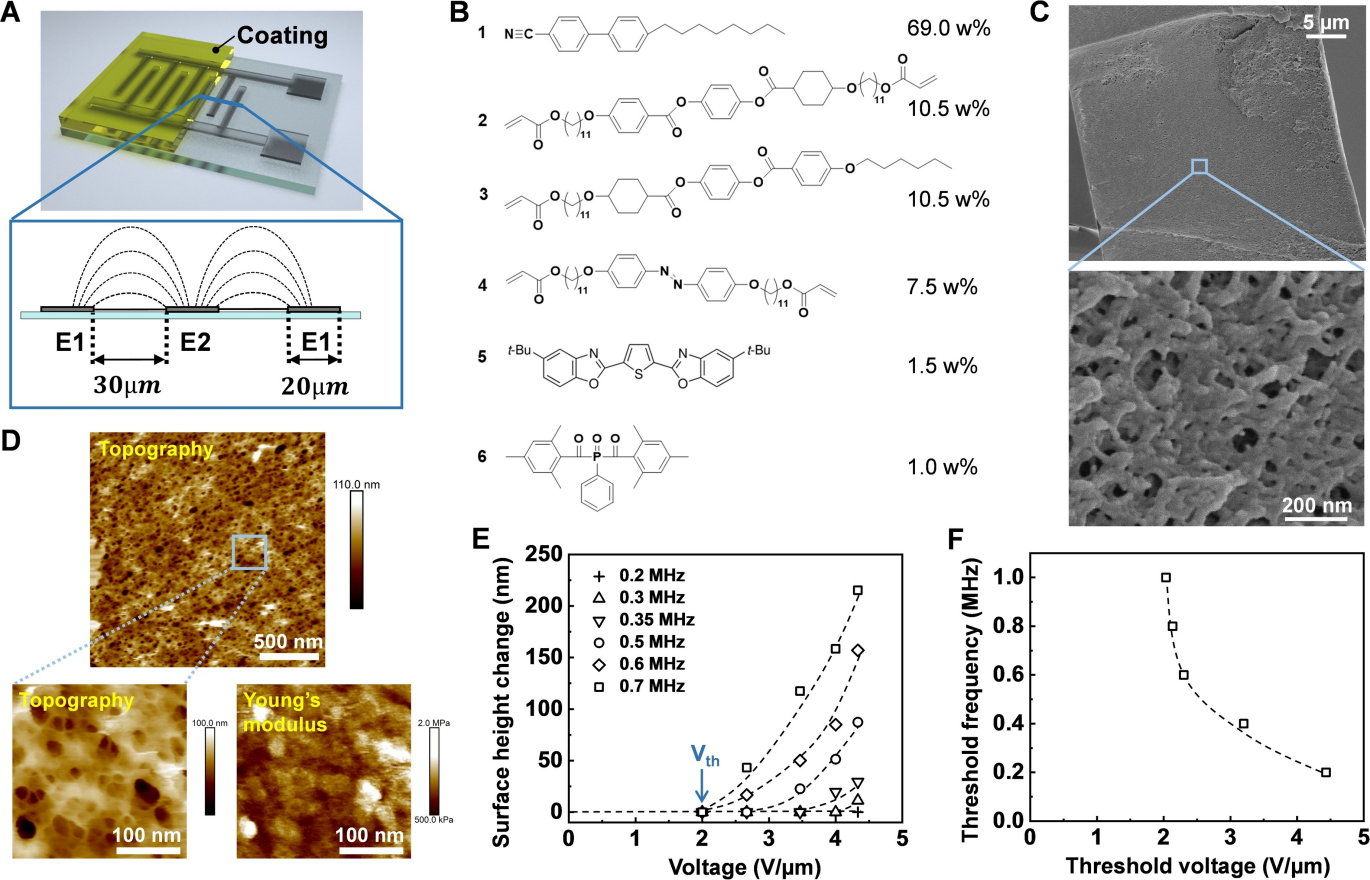
Nanoporous polymer coatings made by liquid crystal networks (LCNs). A) Schematic illustration of the LCN coating on top of the interdigitated electrodes (IDEs). Insert schematically represents the side view of imaginary electric field lines distribution. B) The composition used for fabrication of the coating for 3D‐liquid manipulation. C) Scanning Electron Microscopic images (cross section) and D) Atomic Force Microscopic images (coating surface) of the nanoporous LCN membrane after removal of porogen by solvent extraction. E) Surface height change as a function of voltage at various frequencies. F) Threshold frequency decreases with increasing threshold voltage.

For the experiment, we employed the monomers shown in Figure 1B to fabricate the LCN coatings by photopolymerization in the aligned state. In order to make LCNs light‐responsive, we incorporated the azobenzene derivative **4** as photoswitch. We chose 4‐octyl‐4′‐cyanobiphenyl, viz. 8CB, as porogen, which supports the alignment and functions as transporting liquid. Reactive mesogen **2** and **3** are used to establish the desired smectic phase with homeotropic alignment. To better track liquid displacement, we added a fluorescent dye, 2,5‐bis(5‐*tert*‐butyl‐benzoxazol‐2‐yl)thiophene (**5**), to the liquid crystal mixture prior to photocuring. This mixture was subjected to UV polymerization,^[8]^ during which the porogen phase separated from the polymer and eventually the porous polymer network was obtained. A UV‐block filter was used to cut off wavelengths below 400 nm. The detailed fabrication process of the coating is shown in Experimental Procedures (Supporting Information). The absorbance and emission of the polymerized coating is shown in Figure S1. We conducted scanning electron microscopy and atomic force microscopy measurements after removal of porogen by solvent extraction. Microscopic images of the LCN membrane show that the nano‐sized pores are ranging from 10 nm to 100 nm in diameter (Figure 1C, D). The Young's modulus of the LCN coating is around 1 MPa.

Compound **1**, which we use here as the smectic porogen, is the dielectric liquid that we, after the formation of the porous network, employed to study our transport phenomena. Under the AC field, the aromatic core modified with the nitrile group strongly couples to the field due to its large dielectric anisotropy. We first characterized its electric response by giving the sample a voltage and frequency sweep, respectively. We observe that the liquid secretion, as indicated by a reduction of the film thickness, takes place above a voltage of 2.0 V μm^−1^ and a frequency of 200 kHz (Figure 1E). We define the voltage at which the first surface change is observed as threshold voltage (*V*_th_). It appears that *V*_th_ is frequency‐dependent. Similarly, for an adjusted constant voltage, there is a threshold frequency (*F*_th_). With increasing *V*_th_, we can see that the corresponding *F*_th_ decreases (Figure 1F). We anticipate that the lower *V*_th_ for higher *F*_th_ relates to a temperature rise originating from the high‐frequency electric field.

We know from our previous work that liquid secretes globally when we expose the porous LCN coatings to UV light. Under the UV illumination, azobenzene isomerizes from the straight *trans* to the bent *cis* state. Since the azobenzene moiety is covalently bonded to the LCNs, this motion contracts the homeotropically aligned smectic network^[5a]^ and might reduce the compatibility of the liquid within the LCNs.^[5b]^ Consequently, the liquid is repelled from the bulk to the surface of the coating, which can be observed by eye but is visualized even better and more accurate with respect to the onset by the addition of the fluorescent dye (Figure 2A, B, Video S1). The fluorescent dye is homogeneously dispersed in liquid **1**, which emits blue light upon 365 nm‐light irradiation. We estimate that 4.5 % of the UV illumination is sacrificed to enhance the visualization (Figure S2). We relate the secreted liquid to the coating surface height decrease. This secretion process is further quantified by using digital holographic microscopy (DHM). It can be seen from Figure S3 that the liquid secretion occurs above a UV intensity of 2.1 mW cm^−2^, further defined as threshold intensity *I*_th_. We observe that the amount of secreted liquid increases with increasing light intensity.

**Figure 2:**
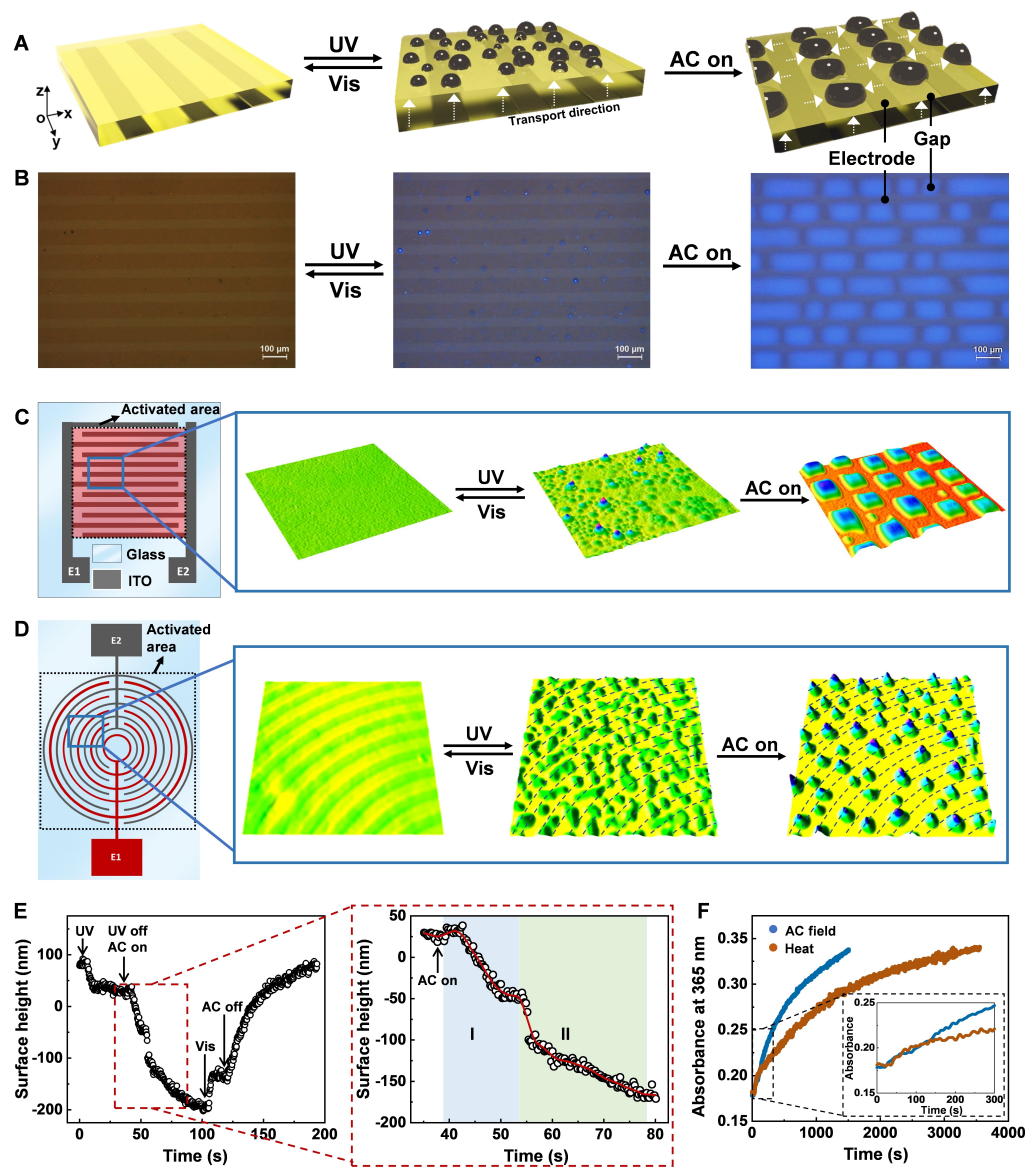
3D‐liquid manipulation. A) Liquid secretion under UV illumination (365 nm, 11 mW cm^−2^) and the subsequent reallocation process driven by the AC field (5.0 V μm^−1^, 200 kHz) at the LCN coating surface, as observed by B) optical microscope and C), D) digital holographic microscope. IDEs with C) square pattern and D) circular pattern are used. E) Surface height change of the LCN coating subjected to sequential light and AC field stimulation. Surface height change occurs between 35 s and 80 s is zoomed in, which was indicated by stage **I** and **II**. Solid red line is eye‐guide. F) Thermal relaxation (at 42 °C) of *cis*‐isomer and the accelerated process under the influence of the AC field. Single point kinetics analysis is performed at the absorbance peak at 365 nm. Insert is the zoom‐in of the first 5 min.

To transport the released dielectric liquid **1** and subsequently collect it at pre‐set locations, we applied an AC field to the IDEs substrate underneath the coating to generate a lateral in‐plane electric field (Figure 1A). We investigated two different types of IDEs patterns, a rectangular pattern and a circular one. The AC field has a voltage of 5.0 V μm^−1^ with a corresponding frequency of 200 kHz. We find that the reallocation process takes approximately 40 s from initiation to completion, as observed by DHM. Looking into more detail (Figure 2C, D, Video S2, S3), we notice that this process is divided into two stages. In stage **I**, immediately after switching on the AC field, the compound **1** experiences phase transition from the smectic phase to the isotropic phase in/at the surface due to a temperature rise. This leads to a slight volume expansion and, consequently, a minor height increase.^[9]^ After eight seconds, the ejected liquid is transported to gaps and aligned with the electric field lines, as indicated by birefringence colour changes (Figure S4, Video S4). In the meantime, not directly observed by optical microscope, the lateral transport of the liquid in the bulk of the coating also occurs. This is suggested by the surface height increase in the gap areas and the corresponding height decrease on top of the electrodes. At the end (stage **II**), the accumulated liquid builds up local pressure, which leads to its ejection at the surface. As a result, the surface height starts to decrease in line with the liquid release (Figure 2E). The reallocated small liquid droplets eventually merge into big ones (Figure 2A, B, C, D). It should be mentioned that the formed big droplets are typically in the shape of a semi sphere when the applied voltage is below 3.0 V μm^−1^ due to surface tension (Figure S5). At a higher voltage, the droplets take a more rectangular shape because of molecular realignment and the electromechanical force exerted.^[10]^ The final reallocated liquid droplets can be clearly visualized by the fluorescent dye, the quantum yield of which slightly increases by 0.8 %, compared to that under the UV irradiation (Figure S2). This is attributed to the realignment of the fluorescent dye and additional liquid release under the AC field.

To better quantify the whole secretion and reallocation process, we calculated the number of droplets that appear at the location of the gap between the electrodes and on top of the electrodes, respectively (Figure 3). Image analysis is carried out using in‐house algorithm by creating a binary map of the IDEs pattern, defining droplets as circles, and tracking the circle centre. The location of droplets is determined by comparing the tracking output with the spatial IDEs distribution. Results are given in Figure 3A (Video S5), in which the IDEs pattern is recognized with blue strips indicated as the electrodes while the yellow ones are the gaps in between the electrodes. Upon UV irradiation, small droplets ranging from 5 μm to 20 μm in diameter appear randomly over the entire surfaces (Figure 3A, C). Gap regions have slightly more droplets, approximately 10 %, than that in electrode regions. This is due to the UV absorption by the indium tin oxide electrodes (Figure S6). We counted that within the analysed region there are approximately 500 droplets, as plotted in Figure 3B. The subsequent AC field transports the small droplets to the gaps, and eventually, the droplets merge into big ones with diameters in the range of 30 μm and 60 μm. This results in the decrease of the total droplets to around 100 counts, in correlation with the conservation of volume. The red circles indicate the sizes of the droplets. They do not represent the actual shapes of them. From close‐up (Figure 3C), we observe that nearly 100 % of droplets are reallocated at gaps. Furthermore, the two stages (lateral transport and secretion) described in Figure 2 are reflected in Figure 3C.

**Figure 3:**
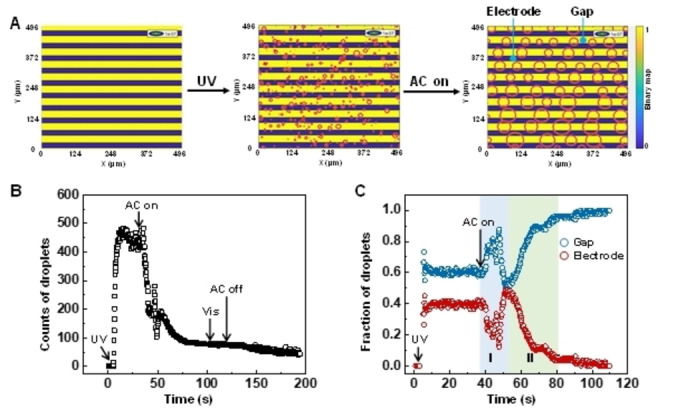
Image analysis of the process of liquid secretion and reallocation, as manifested by A) droplets‐tracking and B) droplet counts over the entire coating surface. C) Fraction of droplets appeared in gap and electrode regions. Stage I and II are highlighted.

We also analysed the influence of the AC field on the isomerization of azobenzene moiety. Results show that the AC field stimulates the *cis*‐to‐*trans* back relaxation. We estimated that the presence of the field converted approximately an extra 13 % of the *cis*‐isomer into its *trans‐*form, as calculated by the increased absorbance of isomerized *trans* over the absorbance at the equilibrium (Figure 2F). We ascribe this to field‐supported electrochemical reduction of the *cis*‐azobenzene.^[11]^ Despite the potential field‐stimulated back‐isomerization, the *cis* conversion is around 90 % at the photostationary state under the condition of combined UV light and electric field. We further observed that the AC field increases temperature (Figure 4D) by 1.2 °C on top of photothermal effect (Figure 4E, F). This is likely because, under the influence of the AC field, UV absorption of azobenzene moiety is slightly enhanced by its reorientation from initial homeotropic alignment to the direction parallel to electromagnetic field of the incident UV light.

**Figure 4:**
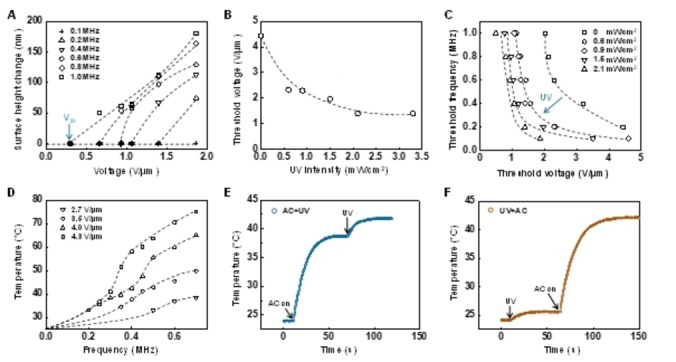
UV‐gated effect. A) Surface height change as a function of applied voltage at various frequencies. B) Decrease in threshold voltage with the increase in UV intensity at the applied frequency of 200 kHz. C) Threshold frequency as a function of threshold voltage with UV illumination at various intensities and in the dark. D) Heat generation upon application of AC field at various frequencies with UV illumination. E), F) Monitored coating temperature over time with sequentially applied AC field and UV light. The applied voltage is of 4.3 V μm^−1^ (300 kHz). Dashed lines are eye‐guide.

In the presence of the AC field, we observe an interesting UV‐gated effect in liquid transport. The threshold voltage *V*_th_ can be lowered by 75 % in the presence of low‐intensity UV light (Figure 1E and Figure 4A), which can be as low as 0.6 mW cm^−2^. Such a low intensity is not sufficient to secrete liquid solely. With this remarkable phenomenon, we studied this gating effect in more detail in terms of threshold voltage and frequency (Figure 4B, C). Figure 4B indicates that we can decrease the threshold voltage by increasing UV intensity. We presume that this unusual UV‐gated secretion effect stems from an increase of pressure of the liquid accumulated underneath the coating surface. This extra pressure is induced by photothermal heating and contraction from photoisomerization of the azobenzene moiety.^[12]^ This result indicates that a minor temperature rise of 3.1 °C is sufficient to significantly increase the local pressure (Figure 4E). Furthermore, when keeping the *V*_th_ constant, a decrease in the *F*_th_ is observed with UV exposure (Figure 4C), as the additional temperature elevation induced by the photothermal effect as well.

Finally, we monitored liquid reabsorption by sequentially irradiating blue light and removing the AC field. The *cis‐*to*‐trans* isomerization of azobenzene generates capillary suction force, which uptakes 27 % of the secreted liquid at the gaps. By stopping the AC field, the rest 73 % of the liquid is reabsorbed into the coating resulting from the elasticity of the polymer network. Eventually, the surface height goes back to its initial value.

In conclusion, we have designed and demonstrated an LCN nanoporous coating for three‐dimensional liquid manipulation, including light‐induced through‐thickness liquid release, field‐driven lateral liquid reallocation, and coalesce through the application of sequential exposure to UV light and the AC field. Furthermore, we have revealed the UV‐gated secretion at the field‐on state, which reduces both threshold voltage and threshold frequency. Future work will focus on generating complex electric fields to position the secreted liquid and we plan to employ modern electrical drive scheme to generate accurate placement and manipulation of the droplets. Moreover, alternative liquids will expand on the applications in various fields. We anticipate that our 3D‐liquid‐manipulating coating can be used for a variety of applications, e.g., cargo/drug release, soft robotics handling, and cell culture manipulating growth factors and nutrient dosing.

## Conflict of interest

The authors declare no conflict of interest.

## Supporting information

As a service to our authors and readers, this journal provides supporting information supplied by the authors. Such materials are peer reviewed and may be re‐organized for online delivery, but are not copy‐edited or typeset. Technical support issues arising from supporting information (other than missing files) should be addressed to the authors.

Supporting Information

Supporting Information

Supporting Information

Supporting Information

Supporting Information

Supporting Information

## Data Availability

The data that support the findings of this study are available from the corresponding author upon reasonable request.
