# Supplementary material for: Light‐ and Field‐Controlled Diffusion, Ejection, Flow and Collection of Liquid at a Nanoporous Liquid Crystal Membrane
Source: Angew Chem Int Ed Engl. 2022 Jul 19;61(38):e202207468. doi: 10.1002/anie.202207468 (PMC9542808; doi:10.1002/anie.202207468)
Supplement: Supplementary file 1 — Supporting Information [file ANIE-61-0-s005.pdf]

Angewandte Chemie
Eine Zeitschrift der Gesellschaft Deutscher Chemiker

Supporting Information

Light- and Field-Controlled Diffusion, Ejection, Flow and Collection of Liquid at a Nanoporous Liquid Crystal Membrane

*Y. Zhan, S. Calierno, J. Peixoto, L. Mitzer, D. J. Broer, D. Liu**

Supporting Information

CONTENTS:

1. Experimental Procedures
2. UV/VIS/NIR absorption and fluorescence emission spectra
3. Fluorescence emission spectra
4. Influence of UV light intensity on liquid secretion
5. Cross-polarized optical microscopy analysis
6. Voltage effect on the shapes of droplet
7. UV/VIS/NIR absorption of ITO
8. References
9. Captions for Video S1 to S4

1. Experimental Procedures

Materials. 4'-Octyl-4-biphenylcarbonitrile (8CB) and molecule **4** were purchased from Synthon. Molecule **2** and **3** were synthesized by Philips Research Laboratory.[1] Photoinitiator Irgacure 819 was purchased from Ciba. Fluorescent dye, 2,5-bis(5-tert-butyl-benzoxazol-2-yl)thiophene, was purchased from Sigma-Aldrich. All chemicals were used as received without further purification. Interdigitated electrodes were kindly provided by the Joint Research Laboratory of Devices Integrated Responsive Materials at South China Normal University.

Coating fabrication. The liquid crystal polymer coating with or without dye was fabricated using the cell technique. Liquid crystal empty cell was constructed by two glass plates: one with interdigitated ITO electrodes (IDE) and the other with conventional plain glass. The IDE plate was treated with 3-(trimethoxysilyl) propyl methacrylate to enhance surface adhesion, and the plain glass was coated with polyimide (SE5661, Nissan Chemical) to provide a homeotropic alignment for liquid crystals.

The liquid crystal monomer mixture solution was made by dissolving components consisting of 69 w% of 8CB, 10.5 w% of molecule **2**, 10.5 w% of molecule **3**, 7.5 w% of molecule **4**, 1.5 w% of fluorescent dye, and 1.0 w% of photoinitiator, in tetrahydrofuran (THF). Prior to cell filling, THF was evaporated. The mixture was then filled in the empty cell with capillary force at 80 °C and isothermal for 20 min. Subsequently, the cell was slowly cooled down to its smectic phase at 32 °C. Finally, the filled cell was exposed to UV light for 90 min for photopolymerization under a UV lamp (Omnicure EXFO S2000) with a UV cut-off filter (<400 nm) placed right below. After curing the glass cell was opened. The coating was released from the glass at the polyimide side.

Characterizations. The thickness of the coating was measured by 3D optical profiler (S Neox, Sensofar). Secretion and reallocation behaviour were monitored by cross-polarized optical microscope (Leica DM2700M) and digital holographic microscope (DHM-R2100). Excitation and emission of the coating were measured by UV/VIS/NIR spectrometer (PerkinElmer 750) and integrating sphere (LMS-100, Labsphere). LED lamps (M365L2 and M455L4-C1, Thorlabs) were used to provide monochromatic light. RF electric field with a sinusoidal wave function was provided by a functional generator (33220A, Agilent). The electric signal from the function generator was amplified by a high-voltage linear amplifier (Falco SystemsWMA-300). The output voltage was measured by an oscilloscope (InfiniiVision DSO-X 3032T, Keysight). The nanoporous structure was measured by scanning electron microscopy (FEI Quanta 3D FEG) and Multimode AFM in the PeakForce Quantitative Nanomechanical Mapping (PF-QNM) mode (Bruker) using HQ: NSC19/No Al (MikroMasch) cantilevers. Image analysis was performed by in-house algorithm. First, the surface profile data obtained from DHM were converted into txt file in which the surface height of each pixel was translated into coordinates. Next, a reference, the initial frame, was used to determine the absolute surface height of droplets. Threshold, binary map, and droplets defining and tracking were then performed successively.

2. UV/VIS/NIR absorption and fluorescence emission spectra

We measured the absorbance and emission of the dye-doped coating by using UV/VIS/NIR spectrometer. The absorbance peak at 365 nm and 380 nm in the excitation state are ascribed to *trans*-isomer of azobenzene moiety and fluorescent dye, respectively.

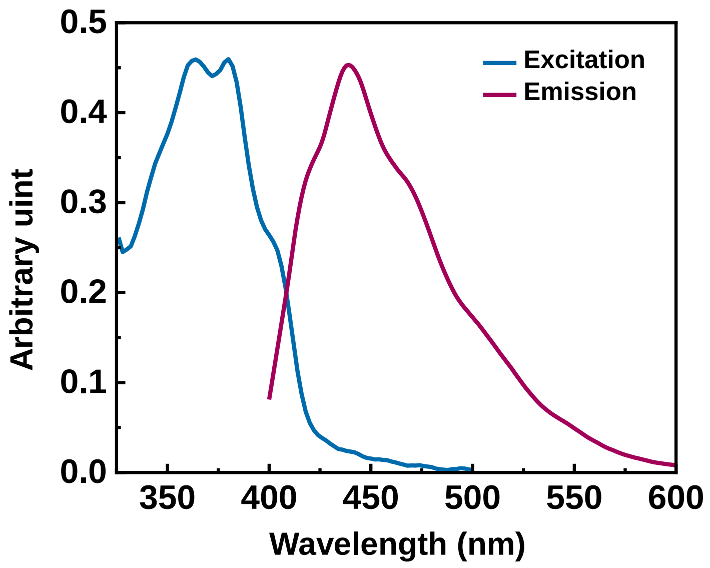

Figure S1. Excitation and emission spectra of the dye-doped coating. Emission is measured upon UV light exposure at 365 nm.

3. Fluorescence emission spectra

The excitation of the fluorescent dye is shown in Figure S2. The measurement is performed in the dyed coating. The maximum absorbance is in the range of 360 nm to 375 nm. Under the incident light at 365 nm, the emission is peaked at 440 nm. We estimate the UV illumination sacrificed to enhance the visualization by calculating our fluorescent emission yield over the average emission yield independent of the host as reported.[2] Result indicates that roughly 4.5% of the UV irradiation is absorbed by the fluorescent dye to improve the visualization.

The quantum efficiency is determined by calculating emitted photons over absorbed photons following the Two Measurement Approach[3] using equation $QE = \frac{P_c}{L_a - L_c}$, where L_a is the number of photons in the excitation wavelength at 365 nm measured in the integrating sphere without sample, L_c is the number of photons in the excitation wavelength at 365 nm measured in the sphere with sample, upon illumination of the incident UV light at 365 nm, and P_c is the number of photons in the emission wavelength at 440 nm with the sample present in the sphere in the path of the incident light beam. Results of measurements are shown in Figure S2. Here we estimate fluorescence quantum efficiency to be 2.6% without the electric field and 3.4% with the field, which was calculated as the emitted photons over absorbed photons.

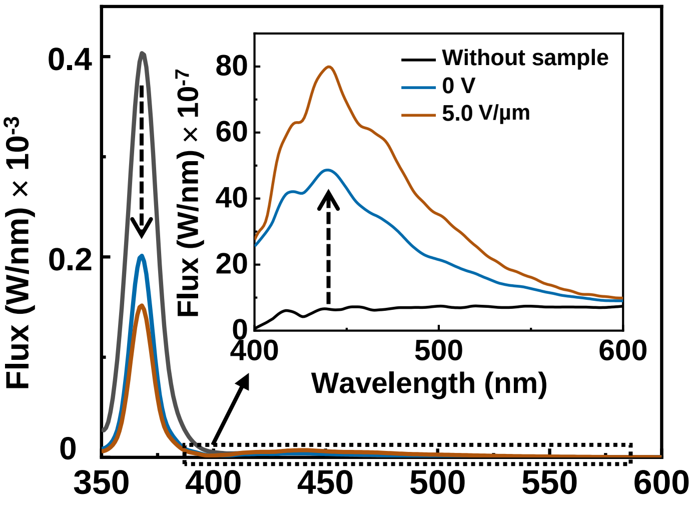

Figure S2. Emission spectra measured without sample (black line) and the measurement with sample at 0 V (blue line) and 5.0 V/µm (orange line). The excitation wavelength used is 365 nm.

4. Influence of UV light intensity on liquid secretion

We correlated the quantity of the secreted liquid to the surface height change, assuming that the unoccupied volume is filled by the polymer after the liquid is repelled. Figure S3 indicates that with increasing UV light intensity, more liquid is secreted. The threshold intensity of liquid secretion is at 2.1 mW/cm^2.

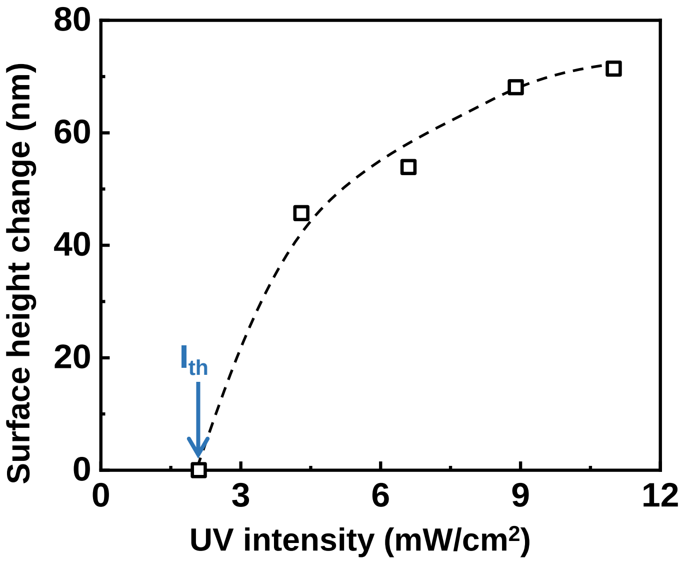

Figure S3. Surface height change as a function of UV intensity. I_{th} indicates threshold intensity for triggering liquid secretion in the absence of an electrical field.

5. Cross-polarized optical microscopy analysis

We can see from cross-polarized optical microscope images that the UV-secreted liquid barely displays birefringence colour, as expected. Upon applying the AC field, the birefringence colour appears, indicating that the secreted droplets are aligned by the electric field. Furthermore, we can see that the liquid is transported and collected at gaps.

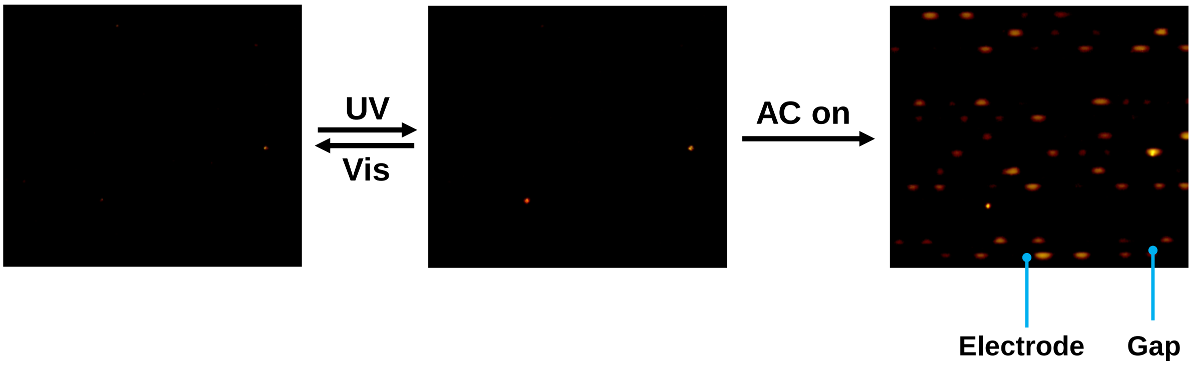

Figure S4. Cross-polarized optical microscopic images showing birefringence color appearance under sequential exposure to UV light and AC field. A yellow light filter is used to block wavelengths below 600 nm to prevent early relaxation of azobenzene *cis*-isomer caused by white LED light from optical microscope.

6. Voltage effect on the shapes of droplet

We observed that with increasing voltage from 2.5 V/µm (V_0) to voltage above 3.0 V/µm (V_1, V_2), the shape of droplets transits from semi sphere to rectangular shape.

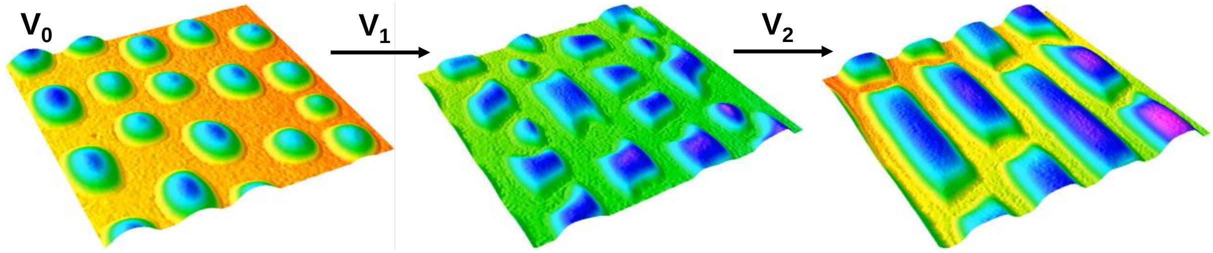

Figure S5. 3D surface profile of the secreted liquid droplets analysed by DHM. The applied voltage $V_2 > V_1 > V_0$.

7. UV/VIS/NIR absorption of ITO

The absorbance of glass with and without ITO coating is measured by UV/VIS/NIR spectrometer. Results show that the absorbance at 365 nm of ITO glass is 10% lower than that of plain glass.

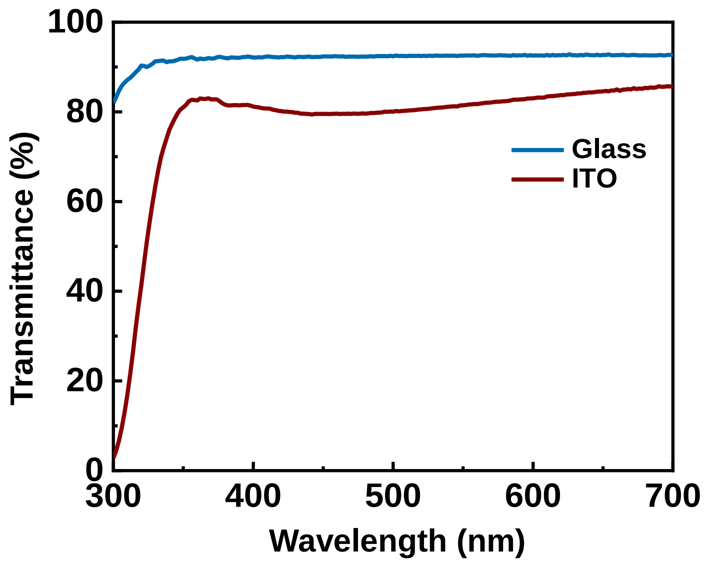

Figure S6. Transmittance spectra of plain glass and ITO coated glass.

8. References

1. J. Lub, E. Peeters, D. J. Broer (Philips Electronics Company), *US007763330B2*, 2010.

2. Fourati, M. A., Skene, W. G., Bazuin, C. G. & Prud'homme, R. E. *Photophysical Study of BBT: A Potential Fluorescent Probe for Polymers*. *Polym. Eng. Sci*. **104**, 182-183 (2011).

3. Leyre, S. *et al.* Absolute determination of photoluminescence quantum efficiency using an integrating sphere setup. *Rev. Sci. Instrum.* **85**, 123115 (2014).

9. Captions for Video S1 to S4

Video S1.

Optical microscopy movie of liquid secretion and subsequent liquid reallocation under sequential UV light irradiation and electric field application.

Video S2.

DHM movie of liquid secretion and subsequent liquid reallocation under sequential UV light irradiation and electric field application.

Video S3.

Cross-polarized optical microscopy movie of liquid secretion and subsequent liquid reallocation under sequential UV light irradiation and electric field application. Electric field controlled centring of dielectric liquid **1** at gaps.

Video S4.

Analysed video of droplet tracking in the process of liquid secretion and reallocation.
